# A cross-sectional study on the assessment of adherence to cardiovascular medications in Sudan heart center

**DOI:** 10.1371/journal.pone.0315672

**Published:** 2025-01-30

**Authors:** Adil A. Mahmoud, Ali Awadallah Saeed, Asim Ahmed Elnour, Osama Nasreldin E. M., Vineetha Menon, Semira Abdi Beshir, Sami Fatehi Abdalla, Abuelnor Mohammed, Mohamed Baraka, Fahad T. Alsulami, Yousef Saeed Alqarni, Nadia Al Mazrouei, Khalid Awad Al-Kubaisi, Israa Yousif El Khidir, Kishore Ganana, Abdulla Al Amoodi

**Affiliations:** 1 Faculty of Clinical and Industrial Pharmacy, Department of Pharmacy Practice, National University-Sudan, Khartoum, Sudan; 2 Faculty of Clinical and Industrial Pharmacy, Department of Pharmacology, National University-Sudan, Mycetoma Research Center, Khartoum, Sudan; 3 Program of Clinical Pharmacy, College of Pharmacy, Al Ain University, Abu Dhabi campus, Abu Dhabi, United Arab Emirates; 4 AAU Health and Biomedical Research Center, Al Ain University, Abu Dhabi, United Arab Emirates; 5 Faculty of Clinical and Industrial Pharmacy, Department of Pharmacy Practice, National University-Sudan, Khartoum, Sudan; 6 Department of Pharmacy Practice, College of Pharmacy, Gulf Medical University, Ajman, UAE; 7 Department of Clinical Pharmacy and Pharmacotherapeutics, Dubai Pharmacy College for Girls, Dubai, United Arab Emirates; 8 Clinical Department, College of Medicine, University of Al Maarefa, Riyadh, Saudi Arabia; 9 Department of Basic Medical Sciences, College of Medical, Dar Al Uloom University, Riyadh, KSA; 10 Department of Histology and Embryology, School of Basic Medical Sciences, Tongji Medical College, Huazhong University of Science and Technology, Wuhan, People’s Republic of China; 11 Pharmacy Department, Associate Professor of Clinical Pharmacy, Fatima College of Health Sciences, Abu Dhabi, United Arab Emirates; 12 Clinical Pharmacy Department, College of Pharmacy, Al-Azhar University, Cairo, Egypt; 13 Clinical Pharmacy Department, College of Pharmacy, Taif University, Taif, Saudi Arabia; 14 Department of Pharmacy Practice, College Of Pharmacy, Imam Abdulrahman Bin Faisal University, Dammam, Saudi Arabia; 15 Faculty of Pharmacy, Department of Pharmacy Practice and Pharmacotherapeutics, University of Sharjah, Sharjah, United Arab Emirates; 16 Department of Pharmacy Practice and Pharmacotherapeutics, College of Pharmacy-University of Sharjah, Sharjah, United Arab Emirates; 17 Department of Clinical Pharmacy & Pharmacy Practice, (Ph.D., MSc, B Pharm), College of Pharmacy, Najran University, Najran, Kingdom Saudi Arabia; 18 Ambulatory Healthcare Services, Academic Affairs, Abu Dhabi Health Services (SEHA), Abu Dhabi, UAE; UCSI University, MALAYSIA

## Abstract

**Background:**

Non-adherence to cardiovascular medications is a global problem with clinical, economic, and humanistic consequences. Investigation of this problem may open the road for proper management of cardiovascular diseases.

**Objective:**

Our objectives were to assess the level of adherence to, and to examine factors influencing adherence to, cardiovascular medications in subjects visiting a heart center in Sudan.

**Methods:**

We have conducted a cross-sectional study that assessed adherence to cardiovascular medications among subjects visiting outpatient cardiac clinics in a heart center-Khartoum State, Sudan. The validated Adherence to Refills and Medication Scale (ARMS) tool was used to assess the level of medication adherence. A score of >16 was used as a cut-off point to categorize surveyed patients into non-adherent (e.g., 17–48) and adherent (e.g., 12–16) in ARMS.

Descriptive statistics (frequencies and percentages) and inferential tests such as One-Way ANOVA and Binary regression were used for data analysis.

**Results:**

A total of 255 subjects were enrolled in this study. Slightly more than half the respondents were males (54.5%) and their ages ranged between (51–60 years), and have no insurance coverage (58%). Most of the subjects were married (60.4%), were from Khartoum-State (67.8%), and were unemployed (77.6%). About 39.6% had primary education while 34.5% had secondary (34.5%) education. Diabetes (56.1%) and hypertension (45.5%) were common among the study population. The results showed a high non-adherence prevalence rate (97.6%). The cited reasons for non-adherence include forgetfulness and the costs of refilling medications. Education and age were associated with the level of adherence. Those with high non-adherence behaviors were the more educated, younger and old patients, those not employed, and those having comorbid diseases.

**Conclusion:**

The results generally showed a high level of non-adherence to cardiovascular medications, necessitating interventions to support patients’ adherence.

## Introduction

The World Health Organization (WHO) adherence project has adopted the following definition of adherence to long-term therapy: The person’s behavior in taking medication extent, following executing lifestyle changes and a diet, corresponds with agreed recommendations from a healthcare provider [1]. Cardiovascular diseases (CVDs) are a group of disorders of the heart, which include coronary artery disease, cerebrovascular disease (stroke), peripheral vascular disease, and congenital heart diseases. It is considered the first cause of death globally (Centers for Disease Control and Prevention [CDC], 2013) [2].

The WHO published a report in 2003 that, in developed countries, adherence rates are about 50% [3]. Adherence is a key factor influencing all pharmacological therapies’ effectiveness, particularly for chronic disease medications. In the United States, 33 to 69 percent of all medication-related hospital admissions are due to poor medication adherence [4]. A large-scale meta-analysis on 376,162 patients from 20 studies, estimated adherence to medication for the prevention of CVDs to be 57% (95% CI 50–64%) [5], these medications are prescribed for a range of long-term conditions including hypertension, dyslipidemia, and angina, among the most prescribed drugs. A study was conducted in Saudi to assess the adherence of heart failure patients using the Morisky -4 scale (MMAS-4) [6]. In Raffaa’s study [6], more than half of the patients had poor medication adherence, and only 7.3% had high adherence. Forty-nine percent forgot their medication at least once, and 34.4% had problems taking it. Another study from Saudi Arabia evaluated medication adherence among patients with chronic disease using the Refills and Medication Scale (ARMS). The results indicated a high percentage of non-adherence (96.62%), 51.9% were missing their medications when getting better, 72.2% forgot to take their medications, 59.2% forgot to take their medications more than once, 51.9% stopped their medications they got better, and 51.7% skipped their dose skipping before doctor visit [7]. A similar study from Latvia that assessed medication adherence levels among patients with hypertension, showed that non-adherence rate of 46.20% where patients with the oldest age and longer duration of disease were more adherent [8].

In a study from Saudi Arabia investigating medication adherence among hypertensive patients attending different primary health centers. The results showed that more than one third (36.3%) were showing high commitment, and the remaining (63.7%) were either in the low or medium range. Adherence was significantly related to age, being married, living in rural areas, and with income of 500 to 7000 Saudi Riyals [9]. In another study about predictors of medication adherence and blood pressure, control among hypertensive patients, the results were has shown that (54%) were non-adherent. Age, gender, being above 65, and being with diabetes were considered independent predictors of medication adherence. The number of patients investigated was 204 [10].

In Sudan, there are fewer studies in the field of medication adherence to chronic diseases, which necessitates investigation of this among patients, especially cardiovascular patients, due to the complexity and diversity of the disease. In a study done at a Sudanese teaching hospital, which evaluated adherence problems to secondary prevention medications among artery disease patients (210 patients), the results showed a 60.7% adherence rate (11). In Elhasan’s study, education level, age, and sex did not affect medication adherence rate [11]. Another study regarding compliance with treatment and quality of life of heart failure patients showed a 75% adherence rate [12].

In a multi-centered study conducted at Khartoum in three cardiac centers, 433 patients were enrolled, and a Morisky scale-8 was used for measuring adherence. This study revealed 49% optimal adherence and 51% poor adherence among patients registered. Respondents with a high level of education and those taking five or more medications were found significantly related to more non-adherents to drug use [13].

## Objectives

The current study’s general objective was to assess medication adherence among CVD patients. The specific objectives were to underline adherence problems, the prescribing pattern of cardiovascular medications, to examine factors influencing adherence to, cardiovascular medications, and to study the correlation between adherence and Sociodemographic characteristics (sex-age-education, etc.) and disease factors (type and duration).

## Methods

The current study design was a cross-sectional Hospital based study conducted, at the Sudan Heart Center-Khartoum State. This was a specialized center, with a capacity of 55 beds belonging to the Medical Service Administration, Ministry of Defense, which also provides services for civilians. According to the 2017 report [14], there were 11 cardiac consultants, and the center underwent 133 surgeries. The study populations were cardiovascular patients who attended the outpatient clinic and who gave their verbal consent by asking the participant a series of questions recorded on notes to participate in the study in period between 12 September 2022 to 30 December 2022. Cardiac patients 18 years of age and older taking at least one medication were interviewed by one pharmacist, while patients coming for surgery were excluded. Convenient samples from patients attending the outpatient clinic were chosen.

### Study instrument and data collection

The research utilized validated adherence tool ARMS [15] in addition to a questionnaire to collect relevant data. Data on Sociodemographic characteristics (gender, age, residence, employment, marital status, education, and insurance), patient clinical information (type of cardiac disease, duration of the illness, and comorbidities)-, medication-related information (number of medications, names, and dose regimen used), level of adherence to medication and drug-related problems encountered during treatment were collected. The researchers did a structured interview for the patients, in which information was explained in Arabic.

### The outcome measure

The questionnaire used to assess medication adherence and overall commitment was operationalized using the ARMS, a 12-item self-reported medication adherence scale in English.

Each item is scored using a 4-point Likert scale (1 = never, 2 = sometimes, 3 = most of the time, and 4 = Always). Ten items assessed adherence behavior while two points assessed issues related to cost and preparation for refiling. The ARMS can range from 12 to 48, with higher scores indicating poor adherence. Moreover, a score of >16 was used as a cut-off point to categorize surveyed patients into non-adherent (e.g., 17–48) and adherent (e.g., 12–16).

### Data analysis

All information gathered via a questionnaire was coded into variables. Reliability Statistics for questionnaire validation were conducted using Cronbach’s Alpha test = 0.561 = 56.1%. Both descriptive and inferential statistics involving the T-Test, Analysis of variances (One Way ANOVA) Test, and binary logistic regression were used in data analysis. A p-value of less than 0.05 was considered statistically significant. One-way ANOVA was used for examining mean differences in adherence across categories of predictors. SPSS, version 23.0, was used for data analysis.

### Ethics approval

Ethical clearance was obtained from the Institutional Review Board of the National University-Sudan (No. NU-REC/11-022/9), and approval was obtained from the Training and Research Department of the Sudan Heart Center.

## Results

### Sociodemographic data of the respondents

Male respondents (54.4%) were slightly higher than females. Most of the respondents (81.3%) were in the age range of 51 years or older, most of the respondents were from Khartoum state (67.8%), most of them were unemployed (77.6%), most of them were married (60.4%), and between primary and secondary education (39.6%) and (34.5%), respectively. More than half of the respondents (58%) had no medical insurance coverage [**Table 1**].

**Table 1:** Sociodemographic data of the respondents: (N = 255).

Sociodemographic characteristics		Frequency (%)
**Gender**	Male	139 (54.5)
	Female	116 (45.5)
**Age groups years(years)**		
	31–40	8 (3.1)
	41–50	27 (10.6)
	51–60	141 (55.3)
	≥ 61	79 (31)
**Residence**		
	Khartoum	173 (67.8)
	Outside Khartoum	82 (32.2)
**Employment**		
	Employed	57 (22.4)
	Not employed	198 (77.6)
**Marital status**		
	Single	66 (25.9)
	Married	154 (60.4)
	Widowed	29 (11.4)
	Divorced	6 (2.4)
**Educational level**		
	Illiterate	32 (12.5)
	Primary education	101 (39.6)
	Secondary education	88 (34.5)
	University and above	34 (13.3)
**Medical insurance**		
	Yes	107 (42)
	No	148 (58)

### Patient’s clinical information

Most types of disease encountered are ischemic heart disease (25.5%), pulmonary embolism (24.3%), and heart failure (24.3%). Duration of disease generally ranged between 1–3 years (40%) and 4–9 years (42.7%). More than half of respondents had comorbid disorders such as diabetes (56.1%) or hypertension (45.5%), [**Table 2**]. Most of the respondents were prescribed three or more medications (69.8%). The result displayed in Table 3 shows that most of the medication prescribed was cardio-selective beta-blockers (44.3%), and diuretics. (40.4%), and angiotensin receptor blockers (ARBs), (33.3%) [**Table 3**]. ARMS adherence Scale results: most of the respondents were non-adherent to the treatment (n = 249, 97, 6%). The practice regarding adherence, for most of the time, was as follows: 49.8% forget to take their medications, 40% often decide not to take their medications, 40.4% miss taking their medication due to carelessness, and 44.7% change the dose to suit their needs. The rate for refilling points (2 points): 27.8% put off filling due to cost, and 16.1% plan to refill. The general results indicate a high tendency towards poor adherence, with slightly fewer problems in the last two points of the scale; the effect of cost and planning for refilling. Most of the Means and standard deviations ARMS scale were above 2 (2.36–2.57), mostly related to forgetfulness about taking medications. The last two points regarding refilling were around 2, [**Table 4**].

**Table 2:** Respondents’ clinical information (N = 255).

Patient clinical information	Frequency (%)
**Type of cardiac disease**	
Myocardial infarction	2 (0.8)
Deep vein thrombosis	6 (2.4)
Heart failure	12 (4.7)
Angina	14 (5.5)
Atrial fibrillation	32 (12.5)
Pulmonary embolism	62 (24.3)
Congestive heart failure	62 (24.3)
Ischemic heart disease	65 (25.5)
**Duration of disease (years)**	
1–3	102 (40)
4–9	109 (42.7)
≥ 10	44 (17.3)
**Co-morbidities** Yes	123 (48.2)
No	132 (51.8)
**Comorbid diseases (frequency = 123)**	
Benign prostatic hyperplasia	1 (0.8)
Asthma	1 (0.8)
Hypothyroidism	5 (4.1)
Gout	13 (10.6)
Rheumatoid arthritis	19 (15.4)
Hypertension	56 (45.5)
Diabetes	69 (56.1)

**Table 3:** Medication used by participants for CVS diseases: (n = 255).

Drug class	Dose	Frequency (%)
**Heart disease medication**	** **	
Bisoprolol	7.5 mg	31 (12.2)
Bisoprolol	5 mg	32 (12.5)
Bisoprolol	2.5 mg	50 (19.6)
Candesartan	8 mg	61 (23.9)
Candesartan	16 mg	21 (8.2)
Candesartan + Hydrochlorothiazide	16/12.5 mg	2 (0.8)
Candesartan	32 mg	3 (1.2)
Amlodipine	5 mg	21 (8.2)
Amlodipine	10 mg	23 (9.0)
Lisinopril	2.5 mg	17 (6.7)
Lisinopril	5 mg	25 (9.8)
Amlodipine/Valsartan	10/160 mg	12 (4.7)
Spironolactone	25 mg	33 (12.9)
Spironolactone	50 mg	4 (1.6)
Digoxin	0.25 μg	12 (4.7)
Furosemide	40 mg	103 (40.4)

**Table 4:** ARMS Adherence scale variables (n = 255).

ARMS Adherence scale variables	Never (%)	Sometimes (%)	Most of the time (%)	Always (%)	Mean (SD) Score
How often do you forget to take your medicine?	18 (7.1)	96 (37.6)	127 (49.8)	14 (5.5)	2.54 (0.708)
How often do you decide not to take your medicine?	20 (7.8)	125 (49)	102 (40)	8 (3.1)	2.38 (0.677)
How often do you forget to get prescriptions filled?	25 (9.8)	99 (38.8)	94 (36.9)	37 (14.5)	2.56 (0.858)
How often do you run out of medicine?	27 (10.6)	96 (37.6)	91 (35.7)	41 (16.1)	2.57 (0.884)
How often do you skip a dose of your medicine before you go to the doctor?	19 (7.5)	120 (47.1)	81 (31.8)	35 (13.7)	2.52 (0.822)
How often do you miss taking your medicine when you feel better?	20 (7.8)	116 (45.5)	83 (32.5)	36 (14.1)	2.53(0.831)
How often do you miss taking your medicine when you feel sick?	30	113	98	14	2.38 (0.763)
	11.8	44.3	38.4	5.5	
How often do you miss taking your medicine when you are careless?	24	121	103	7	2.36 (0.69)
	9.4	47.5	40.4	2.7	
How often do you change the dose of your medicines to suit your needs (like when you take more or fewer pills than you’re supposed to)?	20 (7.8)	108 (42.4)	114 (44.7)	13 (5.1)	2.47 (0.714)
How often do you forget to take your medicine when you are supposed to take it more than once a day?	27 (10.6)	125 (49)	79 (31)	24 (9.4)	2.39 (0.801)
How often do you put off refilling your medicines because they cost too much money?	79 (31)	99 (38.8)	71 (27.8)	6 (2.4)	2.02 (0.828)
How often do you plan and refill your medicines before they run out?	86 (33.7)	106 (41.6)	41 (16.1)	22 (8.6)	2 (0.92)

### Sociodemographic characteristics and adherence levels

The results of one-way ANOVA showed that the level of adherence significantly differed between different age groups (P = 0.002), employment status (P = 0.027), and co-morbidities (P = 0.015). Patients aged 41–50 years were the most adherent (mean 26.04) while patients aged 51–60 years (mean 29.09) and above 61 years of age (mean 28.9) were found to be less adherent. In addition, employed patients were more adherent (mean 27.7 ± 2.2) than those not employed. Respondents with co-morbidities were found to be less adherent than respondents without co-morbidities. In our study, the main predictor of adherence is the educational level (adjusted odd ratio 23.6, CI 95%: 1.58–28.5, P = 0.013).

### Comparison between current study and selected relevant studies (2015–2023)

The results of the current study were compared with the international studies regarding, **adherence tool, level of non-adherence, reasons cited for non-adherence, and predictors of non-adherence.** In area of adherence tool, our study used refills and medication scale tools, Nikolic study [16] use refills tool only, versus Self-reported adherence tool [8,17–20], Eight item Morisky’s medication adherence scale tool [8,13,21–27], Four item Morisky’s medication adherence scale tool [28–31], Four item Morisky Green Levine scale [32], Quantitative scale [33,34], Three item Voils medication non adherence screener [35], Drug attitude inventory tool [36], mass spectrometry urine analysis tool [37–40], Primary medication adherence [41], Urine antihypertensive assay tool [42], dried blood spot analysis tool [23], Recall of number of missed doses of medications tool [43], Proportion of days covered tool [44,45], Health Styles 2010 survey questions tool [46], Questionnaire of non-adherence to Medicines of the Qualiaids Team tool [47], and urine toxicology test tool [20]. In level of non-adherence, all studies showed less non adherence level compared to the current study (97.6%) and Kotian study (HTN = 97.5%, Diabetes = 96.5%) [43], non-adherence more than 50% occurred in studies done by Lawson (55.3%) [42], Wallbach (58.1%) [39], Adidja (66.7%) [26], Awad A (51%) [13], Lo SH (55.9%) [46], Santra G (73.6%) [29], Khasal 54.4% [22], Gikunda study (64.1%) [34], Algabbani (57.8%) [24], and Young (72.7%) [20]. While non-adherence less than 50% occurred in other studies [8,16–19,21,23,25,27,30,32,33,35–38,40,41,44–47].

Regarding the reasons for non-adherence in our study were, **changed the dose to suit their needs (44.7%)**, **decided not to take medications (40%),** 5.9% [21], and **forgot to take their medications (49.8%), 8.2%** [21]**, 15.2%** [35]**, 44.4%** [22]**, 26.3%** [36], 85.1% [23], 16.4% [34], 62.7% [18], 49.41% in patients with diabetes, 26.21% in patients with HTN [43], 23.6% [46], 57.9% [29], 57.8% [20], **missed to take their medications due to carelessness 40.4%, 29.6%** [35]**, 34.6%** [29].

Compared to other reasons found e.g. affordability 20.6% [21], 6.6% [35], 85.1% [23], 9% [18], 67.9% [20], 35.1% [46], 54.4% [13], 85.1% [23], 25.6% [22], 50.58% patients with diabetes, 73.78% patients with HTN [43], undesirable effects of medicines 17.1% [21], 10.4% [35], 4.4% [22], 93.6% [23], 5% [46], 0.2% [36], 9% [18], 36.8% [13], 48.6% [29], and 27.8% [20], taking too many medications 20.1% [35], 11.3% [22], lack of access to medicines or health facility (33.5%), never miss a dose (2.4%) [21], inconvenience of taking the medication 85.1% [23], lack of funds (55.7%), lack of time for refills (4.6%), thought they had healed (3.3%) [34], irregular availability of the drugs in their areas (33.4%), lack of pharmacist’s communication with them regarding the instructions and importance of taking the drug regularly (50.0%), lack of physician’s communication with them regarding their illness and the benefit that the medication will provide (40.4%), poly-pharmacy (52.8%) [13], and others [18,20,22,35,36,46].

Predictors of non-adherence in our study were younger [8,30,31,36,42,46] and older age [18,19,22,34,46], more comorbidity [27,39], higher education [13], unemployment (p < 0.05) compared to others predictors in other studies e.g. multiple antihypertensive medications [19,27,30,37–39,42,47], increased number of medications [13,16,22,23,26,42], unaffordability of treatment [13,20,26,30,31,33], lower monthly income [34,36], male gender [30,31], female gender [38,42], Poor knowledge about HTN (p < 0.05) [24,25], alcohol consumption [13,25], issues with remembering the dosing regimen [16,26,30,44], never doing follow-up visits [22,47], inadequate counseling by physician [21,30,34], in one study no predictors were identified using regression analysis [40], and other predictors were found [8,17–21,25–27,31,33–35,39,42,44–46] All the above-mentioned findings in the compared studies to our current study were depicted in [**Table 5**]

**Table 5:** Comparison between current study and selected relevant studies (2015–2023).

**Relevant studies ↓**	**Study setting**	**Study design**	**Country**	**Number of patients**	**Adherence tool**	**Level of non-adherence**	**Reasons cited for non-adherence**	**Predictors of non-adherence**
Current study, A A Elnour, 2024 (Unpublished)	Out-patients	Cross-sectional hospital-based study	Sudan	255	Adherence to refills and medication scale	97.6%	Changed the dose to suit their needs (44.7%), decided not to take medications (40.0%), forgot to take their medications (49.8%), missed to take their medications due to carelessness (40.4%)	Younger and older age, more comorbidities, higher education, unemployment (p < 0.05)
Kim KY, 2023 [18]	Data from Korea National Health and Nutrition Examination Survey	Nationally representative population-based cross-sectional survey	Korea	6,493	Self-reported adherence	3.6%	-	Women Anxiety/depression, immobility (p < 0.05) Men Anxiety/depression, living together (p < 0.05)
Nikolic A, 2023 [19]	Outpatients	Multi-methods approach: Cross-sectional analytical observational study followed by case-control study	Serbia	338	Number of times patients missed to refill monthly prescribed medications in last 12 months according to the pharmacy dataset	26.0%	-	Burden of taking the drug, complex dosing regimen, current poor control of HTN compared to last year, effect of medications, family history of HTN, increased number of medications, issues with remembering the dosing regimen, issues with purchasing a medication, issues with monthly renewal of medications, not using medications in the last 7 days (p < 0.05)
Noreen N, 2023 [20]	Outpatients	Hospital based cross-sectional study	Pakistan	450	Eight item Morisky’s medication adherence scale	37.8%	Affordability (20.6%), do not wish to take medicines (5.9%), forget to take medicines (8.2%), lack of access to medicines or health facility (33.5%), never miss a dose (2.4%), undesirable effects of medicines (17.1%), others (12.4%)	Fewer years of education, inadequate counselling by physician, unaffordability of treatment, uncontrolled HTN (p < 0.05)
Yousuf FS, 2023 [21]	Inpatients	Cross-sectional study	Pakistan	260	Four item Morisky Green Levine scale	35.8%	-	-
Saeed A, 2023 [22]	Outpatients	Comparative cross-sectional study	Pakistan	168	Quantitative scale	Medium adherence = 22.6% Low adherence = 57.1%	-	Hospital affiliation (particularly public tertiary care hospital) (p < 0.05)
Kharmats AY, 2023 [23]	Outpatients	Cross-sectional study	United States	242	Three item Voils medication nonadherence screener	45.0%	Being too busy (32.0%), BP too low (6.7%), cost (6.6%), concern about interaction (5.7%), feeling ill taking BP medications (8.5%), forgetting to take medications while travelling (15.2%), having difficulty remembering to take medications (29.6%), no symptoms of high BP (16.2%), running out of medications (14.2%), side effects (10.4%), taking too many medications (20.1%), worried about taking medications all life (16%)	Black participants (p < 0.05)
Khasal QA, 2022 [24]	Outpatients	Descriptive cross-sectional study	Pakistan	195	Eight item Morisky’s medication adherence scale	54.4%	Disappearance of symptoms (1.5%), effect of financial strain (25.6%), forgetfulness to take medications (44.4%), multiple medications (11.3%), side effects (4.4%), stopping or changing medications because of perception of not working (1.5%), too busy (11.3%)	Age > 60 years, more number of used medications (p < 0.05)
Abbreviations: BP = Blood pressure, HTN = Hypertension; values of p < 0.05 were accepted as significant								

**Relevant studies ↓**	**Study setting**	**Study design**	**Country**	**Number of patients**	**Adherence tool**	**Level of non-adherence**	**Reasons cited for non-adherence**	**Predictors of non-adherence**
Current study, A A Elnour, 2024 (Unpublished)	Out-patients	Cross-sectional hospital-based study	Sudan	255	Adherence to refills and medication scale	97.6%	Changed the dose to suit their needs (44.7%), decided not to take medications (40.0%), forgot to take their medications (49.8%), missed to take their medications due to carelessness (40.4%)	Younger and older age, more comorbidities, higher education, unemployment (p < 0.05)
Abbas H, 2022 [25]	Outpatients	Multi-methods approach: Cross-sectional study followed by observational retrospective cost of illness study	Lebanon	494	Self-reported adherence and Drug attitude inventory	Overall nonadherence = 36.4% Self-reported nonadherence = 30.6% Drug attitude inventory = 9.9%	Complexity (2.3%), don’t need it/don’t think it works (1.8%), side effects (0.2%), simple forgetfulness (26.3%)	Monthly income lower than 1000 USD, never doing follow-up visits, younger age
Sheppard JP, 2022 [26]	Outpatients	Prospective observational cohort design	United Kingdom	191	Self-reported adherence and mass spectrometry urine analysis	Self-reported nonadherence = 9.4% Biochemically determined nonadherence = 0.5%	-	Prescribed more antihypertensive medications (p < 0.05)
Singer AG, 2022 [27]	Pharmacy-based dispensations data from the Manitoba Population Research Data Repository	Retrospective observational study	Canada	200,000 (91,660 prescriptions)	Primary medication adherence (Absence of dispensing record within 90 days of writing a new prescription)	Antibiotics = 17.5% Antidepressants = 13.7% Antihypertensives = 30.3% Benzodiazepines = 15.7% Bisphosphonates = 27.6% Hypoglycemics = 21.2% Lipid lowering agents = 15.2%	-	-
Alalaqi A, 2021 [28]	Outpatients	Cross-sectional study	Iraq	303	Eight item Morisky medication adherence scale and dried blood spot analysis	Morisky medication adherence scale = 18.2% Dried blood spot analysis = 49.2%	Morisky medication adherence scale Financial cost of medications, forgetfulness, inconvenience of taking the medication (85.1%), medication-related side effects (93.6%), patient’ beliefs, poor understanding of disease	Increase in number of medications prescribed, increase in number of tablets of different medications taken by each patient (p < 0.05)
Gikunda C, 2021 [29]	Outpatients	Descriptive design	Kenya	81	Quantitative scale	64.1%	Forgetfulness (16.4%), lack of funds (55.7%), lack of time for refills (4.6%), thought they had healed (3.3%)	Increasing age, lower monthly income, overall model of health system related factors (availability of medicine, health education, physician patient relationship, stock out, quality of health service) (p < 0.05)
Kulkarni S, 2021 [30]	Outpatients	Retrospective study	United Kingdom	174	Mass spectrometry urine analysis	Overall nonadherence = 40.3% Complete nonadherence = 14.4% Partial nonadherence = 25.9%	-	Female gender, increase in number of antihypertensive medications prescribed (p < 0.05)
Algabbani FM, 2020 [31]	Outpatients	Cross-sectional study	Saudi Arabia	306	Eight item Morisky’s medication adherence scale	57.8%	-	Poor knowledge about HTN (p < 0.05)
Abbreviations: HTN = Hypertension; values of p < 0.05 were accepted as significant								

**Relevant studies ↓**	**Study setting**	**Study design**	**Country**	**Number of patients**	**Adherence tool**	**Level of non-adherence**	**Reasons cited for non-adherence**	**Predictors of non-adherence**
Current study, AA Elnour, 2024 (Unpublished)	Out-patients	Cross-sectional hospital-based study	Sudan	255	Adherence to refills and medication scale	97.6%	Changed the dose to suit their needs (44.7%), decided not to take medications (40.0%), forgot to take their medications (49.8%), missed to take their medications due to carelessness (40.4%)	Younger and older age, more comorbidities, higher education, unemployment (p < 0.05)
Abbas H, 2020 [32]	Outpatients	Cross-sectional study	Lebanon	1,497	Self-reported medication adherence	16.3%	Do not need/do not think about it (16.4%), high medication cost (9.0%), interaction with other medications (0.8%), side effects (9.0%), simple forgetfulness (62.7%), others (2.1%)	Divorced/separated, married, and widowed patients, older patients, obese patients, patients with abnormal BP readings, patient who did not believe in the effectiveness of treatment, patients with poor control of stress level, patients who smoked hookah and cigarettes (p < 0.05)
Sarkodie E, 2020 [33]	Outpatients	Cross-sectional study	Ghana	370	Eight item Morisky’s medication adherence scale	10.8%	-	Alcohol consumption, poor knowledge of HTN, poor perception of severity (p < 0.05)
Lawson AJ, 2020 [34]	Outpatients	Retrospective analysis	United Kingdom	300	Urine antihypertensive assay	55.3%	-	Female gender, higher number of antihypertensive drugs, higher median eGFR, higher SBP and DBP, patients prescribe CCB and diuretics, total number of drugs prescribed, younger age (p < 0.05)
Kotian SP, 2019 [35]	Outpatients	Community-based cross-sectional observational study	India	208	Recall of number of missed doses of medications over 2 weeks prior to interview	HTN = 97.5% Diabetes = 96.5%	Difficulty to remember to take daily medication due to work or forgetfulness (49.41% patients with diabetes, 26.21% patients with HTN), lack of money (50.58% patients with diabetes, 73.78% patients with HTN)	-
Wallbach M, 2019 [36]	Inpatients	Prospective cross-sectional study	Germany	62	Mass spectrometry urine analysis	Overall nonadherence = 58.1% Partially nonadherent = 33.9% Fully nonadherent = 24.2%	-	Longer history of HTN, prescribed more general medications and antihypertensive medications, presence of several co‐morbidities (diabetes mellitus, congestive heart failure, chronic kidney disease, depression) (p < 0.05)
Van der Laan DM, 2019 [37]	Outpatients	Observational cross-sectional study	Netherlands	255	SFK software (Proportion of days covered)	42.7%	-	Difficulties with medication use due to forgetting, insufficient knowledge on what to do when a dose is forgotten, having an ambivalent attitude toward medicines (p < 0.05)
Gavrilova, 2019 [8]	Outpatients	Cross-sectional, observational, quantitative, descriptive study	Latvia	187	Eight item Morisky’s medication adherence scale	46.2%	-	Not following the medication prescriptions of their family physician, shorter duration of HTN, younger age (p < 0.05)
Hayes P, 2019 [38]	Outpatients	Cross-sectional study	Ireland	235	Mass spectrometry urine analysis	Partially adherent = 23.8% Fully nonadherent = 2.1%	-	No predictors were identified using regression analysis
Abbreviations: Abbreviations: BP = Blood pressure, CCB = Calcium channel blockers, DBP = Diastolic blood pressure, eGFR = Estimated glomerular filtration rate, HTN = Hypertension, SBP = Systolic blood pressure, SFK software = Dutch Foundation of Pharmaceutical Statistics; values of p < 0.05 were accepted as significant								

**Relevant studies ↓**	**Study setting**	**Study design**	**Country**	**Number of patients**	**Adherence tool**	**Level of non-adherence**	**Reasons cited for non-adherence**	**Predictors of non-adherence**
Current study, A A Elnour, 2024 (Unpublished)	Out-patients	Cross-sectional hospital-based study	Sudan	255	Adherence to refills and medication scale	97.6%	Changed the dose to suit their needs (44.7%), decided not to take medications (40.0%), forgot to take their medications (49.8%), missed to take their medications due to carelessness (40.4%)	Younger and older age, more comorbidities, higher education, unemployment (p < 0.05)
Singh K, 2019 [39]	Outpatients	Retrospective concept-wide association study	United States	8,218	Proportion of days covered	25.4%	-	Asthma/COPD management program, blood pressure medication, case management, assessment of needs at discharge, mood disorders, social determinants of health, coordination of care between physician’s office and patient, documentation of symptoms, home health needs, refractory to treatment, noncompliance, language barrier, presence of drug allergies, management of cardiac arrhythmias with warfarin, neurological disorders, plantar fasciitis instructions, urinalysis, volume depletion and overload (p < 0.05)
Adidja NM, 2018 [40]	Outpatients	Cross-sectional community-based study	Cameroon	183	Eight item Morisky’s medication adherence scale	66.7%	-	Adverse drug effects, financial constraints, forgetfulness, multiple daily doses (p < 0.05)
Asgedom SW, 2018 [41]	Outpatients	Hospital-based cross-sectional study	Ethiopia	280	Eight item Morisky’s medication adherence scale	38.2%	-	Presence of co-morbidities, regular alcohol intake, self-purchasing medications, taking combination of antihypertensive medications (p < 0.05)
Awad A, 2017 [13]	Outpatients	Descriptive cross-sectional study	Sudan	386	Eight item Morisky’s medication adherence scale	51.0%	Bothered by side effects associated with their medications (36.8%), expensive cost of drugs (54.4%), irregular availability of the drugs in their areas (33.4%), lack of pharmacist’s communication with them regarding the instructions and importance of taking the drug regularly (50.0%), lack of physician’s communication with them regarding their illness and the benefit that the medication will provide (40.4%), polypharmacy (52.8%)	High level of education, low- and middle-income levels, patients taking five or more medications daily (p < 0.05)
Butler MJ, 2017 [42]	Patients from JHS	Prospective population-based cohort study	United States	3,558	Self-reported medication adherence	27.1%	-	Older, less likely to be smokers, had fewer years of education, less likely to be employed, more likely to have diabetes, more likely to be taking three or more classes of antihypertensive medication, more likely to be taking loop diuretics and angiotensin receptor blockers (p < 0.05)
Lo SH, 2016 [43]	Outpatients	Cross-sectional correlational study	Hong Kong	195	Four item Morisky’s medication adherence scale	55.9%	-	Younger age, living with family, poor illness perception related to treatment control (p < 0.05)
Abbreviations: COPD = Chronic obstructive pulmonary disease, JHS = Jackson Heart Study; values of p < 0.05 were accepted as significant								

**Relevant studies ↓**	**Study setting**	**Study design**	**Country**	**Number of patients**	**Adherence tool**	**Level of non-adherence**	**Reasons cited for non-adherence**	**Predictors of non-adherence**
Current study, A A Elnour, 2024 (Unpublished)	Out-patients	Cross-sectional hospital-based study	Sudan	255	Adherence to refills and medication scale	97.6%	Changed the dose to suit their needs (44.7%), decided not to take medications (40.0%), forgot to take their medications (49.8%), missed to take their medications due to carelessness (40.4%)	Younger and older age, more comorbidities, higher education, unemployment (p < 0.05)
Tong X, 2016 [44]	Outpatients	Consumer mail panel surveys	United States	1,366	HealthStyles 2010 survey questions	30.5%	Cannot afford the medication (35.1%), don’t think there is need to control blood pressure (27.1%), forgot to take medication (23.6%), ran out of medication (12.5%), do not like side effects from medications (5%), feels okay without medication (4%), other reason (3.9%), don’t know (2%), shares medications with someone else (0.5%), medication is not working (0.3%), thinks should be taking a different medication (0.2%), multiple reasons selected (25.7%)	Age of 18–44 years and 45–64 years, hispanic race, household income < 25,000 USD (p < 0.05)
Santra G, 2015 [45]	Inpatients and outpatients	Observational study	India	280	Four item Morisky’s medication adherence scale	Overall nonadherence = 73.6% HTN = 79.2% CCF = 71.7% IHD = 68.0%	Careless at times to take medications (34.6%), forget to take medications (57.9%), stops taking medications when felt better (37.5%), stops taking medications if it makes them feel worse (48.6%)	-
Bader RJK, 2015 [46]	Outpatients	Cross-sectional multicenter study	United Arab Emirates	250	Four item Morisky’s medication adherence scale	45.6%	-	Being aware of the complications of HTN, cost of medication, forgetfulness, method of identifying medication (by the color or shape of the package vs name), male gender, more children, multiple hospital admissions, multiple antihypertensive medications, patients’ perceived effectiveness of antihypertensive medication, patients’ use of traditional remedies, patients whose last clinic visit was > 1 month ago, patients who did not receive medication education from healthcare providers, patients whose antihypertensive drugs were changed more than once by the physician, patients’ lack of awareness of their physician’s instructions regarding their medication (p < 0.05)
Barreto Mda S, 2015 [47]	Outpatients	Descriptive cross-sectional study	Brazil	422	Questionnaire of non-adherence to Medicines of the Qualiaids Team	42.7%	-	Low educational levels, not regularly attending doctor’s appointments, not have private health insurance, non-Caucasian hypertensive patients, taking more than two antihypertensive medications (p < 0.05)
Abbreviations: CCF = Congestive cardiac failure, HTN = Hypertension, IHD = Ischemic heart disease; values of p < 0.05 were accepted as significant								

**Relevant studies ↓**	**Study setting**	**Study design**	**Country**	**Number of patients**	**Adherence tool**	**Level of non-adherence**	**Reasons cited for non-adherence**	**Predictors of non-adherence**
Current study, A A Elnour, 2024 (Unpublished)	Out-patients	Cross-sectional hospital-based study	Sudan	255	Adherence to refills and medication scale	97.6%	Changed the dose to suit their needs (44.7%), decided not to take medications (40.0%), forgot to take their medications (49.8%), missed to take their medications due to carelessness (40.4%)	Younger and older age, more comorbidities, higher education, unemployment (p < 0.05)
Morrison VL, 2015 [48]	Outpatients	Multinational cross-sectional survey	11 European countries	2,595	Four item Morisky’s medication adherence scale and medication adherence rating scale	Morisky medication adherence (Mean score of nonadherent respondents) Netherlands = 24.1 (18.6–29.5) Germany = 33.2 (27.6–38.8) Austria = 33.7 (28.6–38.9) Wales = 38.1 (32.8–43.4) Belgium = 38.9 (31.8–46.0) England = 41.5 (36.1–46.9) Greece = 50.2 (44.4–55.9) Poland = 57.6 (52.2–63.0) Hungary = 70.3 (65.3–75.3) Medication adherence rating scale Netherlands = 23.86 (23.64–24.16) Germany = 23.47 (23.28–23.75) Austria = 23.25 (23.03–23.56) Wales = 23.46 (23.30–23.77) Belgium = 23.59 (23.50–23.99) England = 23.41 (23.17–23.65) Greece = 22.08 (21.71–22.48) Poland = 18.19 (17.77–19.01) Hungary = 22.88 (22.74–23.26)	-	Morisky medication adherence Being of younger age, being employed, difficulty in borrowing money, high normative beliefs, high perceived barriers, high dosing frequency, low number of medicines, low self-efficacy, low personal control, low concern about illness, males (p < 0.05) Medication adherence rating scale Beliefs in treatment control, greater number of medicines, having low perceived barriers, high self-efficacy, lower level of education, less frequent dosing, low perceptions of illness consequences, older age (p < 0.05)
Young JH, 2015 [49]	Inpatients	Prospective cohort study	United States	187	Self-reported medication adherence and urine toxicology test	Missed medication prior to admission = 72.7% Missed at least one dose of medication in a typical week = 42.8% Running out of medications for a day or more at least three times a year = 32.1% All three nonadherence measures = 19%	Blood pressure pills don’t help (22.9%), can’t afford medications (67.9%), can’t find doctor to prescribe (22.9%), can’t get to a pharmacy (26.7%), experience side effects (27.8%), forget to take medicine (57.8%), take pills too many times per day (16.0%)	Missed medication prior to admission = Uninsured, can’t afford medications Missed at least one dose of medication in a typical week = Insured with medication copay, HTN knowledge, experience side effects, forget to take medicine, blood pressure pills don’t help, take pills too many times per day Running out of medications for a day or more at least three times a year = Uninsured, can’t afford medications (p < 0.05)
Abbreviations: HTN = Hypertension; values of p < 0.05 were accepted as significant								

## Discussions

The general results of this study revealed there is a high prevalence rate (97.6%) of non-adherence to medications among people with CVDs. The results indicated a high tendency towards poor adherence, in most of the 10 points of the ARMS scale, with slightly fewer problems in the last two ends of the scale i.e. the effect of cost and planning for refilling. The cited reasons for non-adherence include forgetfulness and the costs of refilling medications.

Due to Misunderstanding (e.g. the nature of side effects, the time it takes to see results or patient does not understand the need for the medicine), and mistrust to prescribed medicines, the patient cannot take the medicines [50].

Education and age were associated with the level of adherence. Those with high non-adherence behaviors were the more educated, those not employed, and those having comorbid diseases. Adherence problems faced by the patients were decreased patient satisfaction due to higher patient-physician discordance, complex medication regimen, and patient feel better or symptom free. Complex medication regimen may be due to drug administration improper timing, or administration of numerous medications at unusual or frequent times during the day. The patient’s ability to read and understand medication instructions is one of major factor that influences adherence. Patients with low educational levels have difficulty in instructions understanding leading to decrease adherence [51].

Sociodemographic factors such as sex, age, income, gender, level of education may be related to non-compliance [51]. The prescribing pattern of cardiovascular medications, were Furosemide (40.4%), Candesartan 8mg (23.9%), Bisoprolol 2.5 mg (19.6%), Bisoprolol 5mg and 7.5mg (12.5% for each), Lisinopril 5,g (10%) and others heart diseases medications. The drug problems encountered by the respondents decrease adherence and can lead to changing the dose to suit their needs, and the decision not to take medications by experiencing side effects [52]. In a study from Latvia [8], the non-adherence rate was less than in the current study (46.2%), and the oldest age and those respondents with a longer duration of disease were more adherent to their medications. Those results were different from our study, which showed respondents aged 51 years or older were more non-adherent to treatment. The duration of the disease showed that those with a duration of 10 years and more (mean 28.66 ± 3.2) were slightly more adherent than those with a disease of 1–3 years duration. These differences between the two studies may be due to different scales used for measuring adherence behavior. Poor adherence among the elderly is expected because these respondents are more susceptible to forgetting their medications. In our study also, those respondents between 31 to 40 years old showed a non-adherence rate almost like the elderly group (mean 29.5 ± 2.4). Those in the range of 41 to 50 years, were within acceptable adherence, maybe because this group of people take on more responsibility for their health and are actively working people, who have good information about their disease status. Compared to a study from Saudi Arabia that assessed adherence among hypertensive respondents [9], the current study showed poor adherence rates. Similar results to the current study regarding the percentage of those without co-morbidities were obtained (49%), while in the current study (51.8%). This is explained by the fact of the presence of many diseases and many medications can affect drug-taking behaviors among respondents with chronic diseases in general. The results of the current study are also comparable to a study among hypertensive patients [10], which reported 54% of patients were non-adherent to their medications. Further factors such as age above 65 years, gender, and diabetes were predictors of poor adherence. In the current study, patients older than 61 years of age and having co-morbidities were more non-adherent to their medications. In our study, the main predictor associated with adherence is the educational level (P = 0.013). It is interesting to find that educated patients are more non-adherent to their medications. This is explained by the fact that educated persons may be behaviorally distracted by different mental activities, which affect their adherence. In contrast, less educated persons follow doctors’ advice precisely, without changes, compared to knowledgeable patients who may sometimes find a reason for delaying their medications without being anxious.

It is evident that patients with CVDs need further investigation about barriers to medication adherence. These barriers could be due to different factors such as disease-related factors, therapy-related factors, healthcare-related factors, patient-related factors, and social-related factors. Adherence could be improved through several mechanisms including enhanced patient-provider communication, using health messages, adherence-assisting devices, providing patient education, and psychological support [48].

The prescribing pattern of medications in the current study showed that most medications prescribed were cardio-selective Beta–blockers, diuretics, and ARBs. Most of them were given in a single dose or twice a day, and this is considered a reasonable effort from prescribers toward improving patients’ adherence. Three studies conducted in Sudan [11–13] reported adherence rates of 60.7%, 75% and 49% in the previous studies, respectively. The adherence rate is better than our study, which only reported an adherence rate of only 4.6%. The first study [11] had a higher prevalence of hypertension and diabetes, as co-morbidities, in this study, the level of education, age, and gender were not associated with adherence. However, our study reported that education level, and age but not gender influence adherence to treatment. The third study [13] supports our finding that a high level of education is associated with poor adherence to their medications. A recent study from Sudan [49] also showed a high non-adherence rate (97.3%) at baseline, which was improved after clinical pharmacist-led educational intervention, and it was in accordance with the present result. The reason for the higher non-adherence rate in this study is explained by the following: the use of different scales or tools for determining adherence; questionnaire data alone is not sufficient to give a clear picture of commitment unless supplemented by practical measures such as checking of blood pressure, hyperlipidemia, cardiac tests and counting of use of prescribed medications. Prescribing more than three medications (69.8%) could be another reason.

### The implications of the current study

- Healthcare professionals should concentrate on patient categories, such as the elderly, both educated and uneducated, and those with co-morbidities, to improve adherence to medications.
- For subjects who have been recently diagnosed and those who are in the initial post-diagnosis years, it is vital to streamline medication regimes and provide close follow-up.
- Pharmacists can actively engage patients through a variety of communication techniques to improve adherence.
- Future studies should investigate practical ways to gauge adherence and examine obstacles to taking medications as prescribed.

The current study emphasizes the need of focused treatments and the involvement of pharmacists in improving medication adherence for improved patient outcomes.

### Limitations

The study includes several limitations that prevent generalizing the study’s findings to the Sudanese community at large. There is a need for a larger multicenter study to improve the generalizability of the results. Additionally, the study did not investigate ways to increase adherence.

## Conclusions

The incidence of non-adherence was disturbingly high at 97.6%, with forgetfulness being the main cause, followed by elements like cost and prescription refilling. It is interesting to note that non-adherence was more common in older and younger patients alike, educated people, people with co-morbid conditions, and people who were unemployed. The prescription pattern showed a preference for diuretics, ARBs, and cardio-selective B-blockers. According to patient input, no drug-related issues, such as side effects or low doses, have been noted. Future studies are needed to describe medication-taking behavior in a larger scope beyond the imperfect and uninformative descriptions of adherence terms.

## Abbreviations

ARBs: Angiotensin receptor blockers
MMAS-4: Morisky -4 scale
WHO: World Health Organization
CVDs: Cardiovascular diseases
CDC: Centers for Disease Control and Prevention
