# Correction: A cross-sectional study on the assessment of adherence to cardiovascular medications in Sudan heart center

**DOI:** 10.1371/journal.pone.0325308

**Published:** 2025-05-27

**Authors:** Adil A. Mahmoud, Ali Awadallah Saeed, Asim Ahmed Elnour, Osama, Nasreldin E. M., Vineetha Menon, Semira Abdi Beshir, Sami Fatehi Abdalla, Abuelnor Mohammed, Mohamed Baraka, Fahad T. Alsulami, Yousef Saeed Alqarni, Nadia Al Mazrouei, Khalid Awad Al-Kubaisi, Israa Yousif El Khidir, Kishore Ganana, Abdulla Al Amoodi

The initials of the tenth author are indexed incorrectly in PubMed. The correct initials are: Alsulami FT.
